# Markers of Atrial Cardiopathy in Severe Embolic Strokes of Undetermined Source

**DOI:** 10.3389/fcvm.2022.903778

**Published:** 2022-06-20

**Authors:** Maurizio Acampa, Alessandra Cartocci, Carlo Domenichelli, Rossana Tassi, Francesca Guideri, Pietro Enea Lazzerini, Giuseppe Martini

**Affiliations:** ^1^Stroke Unit, Department of Emergency-Urgency and Transplants, Azienda Ospedaliera Universitaria Senese, “Santa Maria alle Scotte” General Hospital, Siena, Italy; ^2^Department of Medical Biotechnology, University of Siena, Siena, Italy; ^3^Department of Medical Sciences, Surgery and Neurosciences, University of Siena, Siena, Italy

**Keywords:** ischemic stroke, ESUS, atrial cardiopathy, atrial fibrillation, cardioembolism, ECG, P-wave-dispersion

## Abstract

**Background and Purpose:**

The current definition of embolic strokes of undetermined source (ESUS) seems to be too broad, including strokes due to heterogeneous mechanisms, such as atrial cardiopathy and other occult cardiac conditions, aortic arch plaques, and non-stenosing atherosclerosis, that can be differently associated with clinical stroke severity at the time of presentation. The aim of our study was to assess the possible association between neurological deficit severity and presence of markers of atrial cardiopathy in ESUS.

**Methods:**

We retrospectively reviewed the medical records of a cohort of 226 ESUS patients (105 M, 121 F), that were divided into two groups according to the severity of neurological deficit (99 mild strokes with NIHSS ≤ 5 and 127 severe strokes with NIHSS >5). The following indices of atrial cardiopathy were evaluated: P wave dispersion, P wave max, P wave min, P wave mean, P wave index, P wave axis, left atrial size.

**Results:**

Patients with severe ESUS were significantly older (74 ± 12 vs. 67 ± 14 years, *P* < 0.001) and female sex was prevalent (67 vs. 36%, *P* > 0.001); they had higher values of P-wave-dispersion (51 ± 14 vs. 46 ± 13, *P* = 0.01), P-wave-max (131 ± 20 vs. 125 ± 15 ms, *P* = 0.01), P-wave-index (16 ± 5 vs. 15 ± 5 ms, *P* = 0.01), left atrial size (20 ± 6 vs. 18 ± 4 cm^2^, *P* = 0.01), left atrial volume index (31 ± 14 vs. 27 ± 11 ml/m^2^, *P* = 0.04), in comparison with mild ESUS. An abnormal P wave axis was detected more frequently in severe ESUS (21 vs. 9%, *P* = 0.01). Furthermore, multivariate logistic regression showed that age (OR = 1.21 for each 5-year increase, 95% CI 1.09–1.35), sex (OR = 3.24 for female sex, 95% CI 1.82–5.76) and PWD (OR = 1.32 for each 10-ms increase, 95% CI 1.07–1.64) were the best subset of associated variables for severe ESUS.

**Conclusions:**

Our findings shed light on specific clinical characteristics of severe ESUS including the presence of atrial cardiopathy that could play a pathogenic role in this subgroup of patients. Searching for atrial fibrillation in these patients is especially important to perform the most appropriate therapy.

## Introduction

Embolic strokes of undetermined source (ESUS) represent 17% of ischemic strokes (1). Even if embolism is presumably the underlying mechanism of these strokes, the current definition of ESUS seems to be too broad (2), including heterogeneous causes, such as occult cardiac conditions (atrial cardiopathy, patent foramen ovale), aortic arch plaques, or non-stenosing atherosclerosis of large cervical and intracranial arteries. These different possible mechanisms have suggested a reexamination of the ESUS concept (3), with the goal of improving specificity for detecting patients with a cardioembolic cause, in order to provide a more appropriate therapy for secondary stroke prevention.

Although clinical characteristics alone cannot reliably classify the underlying cause of ischemic stroke (4), higher National Institutes of Health Stroke Scale (NIHSS) score is usually associated with cardioembolic etiology (5). Furthermore, patients with severe ESUS (NIHSS score >5) have different clinical characteristics and outcome with higher mortality rate in comparison with patients with mild ESUS (6). These findings suggest a possible role of cardioembolic mechanisms (especially due to atrial cardiopathy) in determining severe ESUS.

In this view, we assessed the possible relationships between severity of neurological deficit at the time of presentation and prevalence of markers of atrial cardiopathy in ESUS patients.

## Materials and Methods

The data that support the findings of this study are available from the corresponding author upon reasonable request. We retrospectively reviewed the medical records of a cohort of 226 patients, admitted consecutively to our Stroke Unit (Siena University Hospital) for ESUS from January 1st 2017 to 30th December 2020. The eligibility criteria were based on the definition of ESUS, proposed by the Cryptogenic Stroke/ESUS International Working Group (7). Neurological status at admission was assessed by using the NIHSS score. All patients underwent neuroimaging examination (brain CT with angio-CT scan and/or brain magnetic resonance imaging), extracranial and transcranial arterial ultrasound, transthoracic echocardiography, 12-lead electrocardiogram, 24-h electrocardiogram. The study was approved by the Ethics Committee of the University Hospital of Siena, Italy.

### Markers of Atrial Cardiopathy

#### P Wave Duration and P Wave Dispersion

Simultaneous 12-lead ECG (25 mm/s and 10 mV/cm) was recorded by means of commercially available imaging system (Cardioline ECT WS 2000, Remco Italia, Vignate-Milano, Italy) in all subjects in supine position (during spontaneous breathing) at the time of admission. Paper-printed ECGs were scanned and digitized in order to achieve greater precision in detecting and measuring P waves (8); onscreen measurement of P wave duration was made by a single observer (M.A.), that had no knowledge about the severity of ESUS, by means of Adobe Photoshop CC 2017 software. P wave duration was measured from the beginning of the P wave deflection from the isoelectric line to the end of the deflection returning to isoelectric line in all simultaneous 12 leads. Our measurement method has been reported in our previous study (9), that demonstrated acceptable intra observer and inter observer errors in the measurement of P-wave duration in 12-lead ECGs, according to other previous papers (8, 10). The following indices were derived from measurements of each ECG: the maximum P wave duration (P max), the minimum P wave duration (P min), the P wave dispersion (PWD), defined as the difference between P maximum and P minimum, P-wave-mean and P-wave-index (the average and standard deviation of P wave duration across all 12 leads). Normal PWD values were lower than 40 ms (11–13).

#### P-Wave Axis

P-wave axis was determined by measuring the positive or negative P-wave deflections on all six limb leads and then calculating the net direction of electric activity using the hexaxial reference system. Automated analysis of ECG data was conducted including selective averaging to obtain representative durations and amplitudes of ECG components to calculate the frontal P-wave axis. An abnormal PWA was defined as any value outside the range between 0 and 75° (14).

#### Left Atrial Size and Mitral/Aortic Valve Disease

Left atrial size was evaluated by transthoracic echocardiography, measuring left atrial area in four-chamber apical view and left atrial volume index using the biplane area-length method (15). Furthermore, the presence of some minor-risk potential cardioembolic sources, such as mitral and aortic valve calcifications, was also evaluated.

#### Non-stenotic Carotid Plaques

According to the methods previously described (16), we evaluated the possible presence of non-stenotic (<50% diameter stenosis) atherosclerotic carotid artery plaques in all patients.

### Statistical Analysis

Statistical analysis was performed using the GraphPad Instat computer software (version 3.06 for Windows, GraphPad Software Inc., La Jolla, CA, USA). All results were presented as mean ± SD values. Normal distribution of quantitative variables was preliminary tested using the Kolmogorov–Smirnov test. Patients were dichotomized into two groups according to the severity of neurological deficit evaluated by NIHSS score: mild ischemic stroke was defined as presenting NIHSS score of 0–5 and severe ischemic stroke as presenting NIHSS score of 6–41. We also performed a sample size and power analysis to define the number of subjects to include in the ESUS cohort: a sample size of 226 was estimated considering a chi-squared test, with alpha of 0.05, power of 0.95 and a medium effect size of 0.25.

Mann-Whitney test was performed to compare age, electrocardiographic and echocardiographic markers of atrial cardiopathy in patients with mild stroke and severe stroke. The two-sided Fisher's exact test was performed to evaluate statistical correlation between categorical variables in both groups of patients. To determine the factors associated with severe ESUS a multivariate logistic regression was performed (with the stepwise procedure based on Akaike's criterion) and corresponding Odds Ratio (OR) and their confidence intervals (CI) were calculated. A *P* value below 0.05 was considered statistically significant.

## Results

We enrolled 226 patients with ESUS (105 males, 121 females). Patients were divided into two groups according to the severity of neurological deficit: 99 patients with mild stroke (NIHSS ≤5) and 127 with severe stroke (NIHSS >5).

In severe ESUS the average age was higher (74 ± 12 vs. 67 ± 14 years, *P* < 0.001) and female sex was prevalent (66 vs. 36%, *P* < 0.001).

Both groups were similar in terms of other cardiovascular risk factors (Table 1).

**Table 1:** Characteristics of ESUS patients with mild and severe neurological deficit.

	**Mild ESUS** **(NIHSS ≤5)** **(*n* = 99)**	**Severe ESUS** **(NIHSS >5)** **(*n* = 127)**	***P*** **-Value**
Age (years)	67 ± 14	74 ± 12	**0.0001**
Women/men	36:63	85:42	**0.0001**
**Cardiovascular risk factors**			
Hypertension, *n* (%)	52 (52%)	82 (65%)	0.07
Diabetes mellitus, *n* (%)	25 (25%)	21 (17%)	0.1
Hypercholesterolemia, *n* (%)	38 (38%)	47 (37%)	0.8
Previous CAD, *n* (%)	7 (7%)	12 (9%)	0.6
Previous stroke, *n* (%)	7 (7%)	10 (8%)	1
Current smoking, *n* (%)	24 (24%)	25 (20%)	0.4
**Minor-risk potential embolic sources**, ***n*** **(%)**			
Mitral valve calcifications	22 (22%)	51 (40%)	**0.004**
Aortic valve calcifications	33 (33%)	54 (42%)	0.1
Non-atrial fibrillation atrial dysrhythmias	1 (1%)	1 (0.7%)	1
Hypokinetic/akinetic left ventricle	2 (2%)	5 (3%)	0.4
Moderate-to-severely dilated left atrium	1 (1%)	10 (7%)	**0.02**
Atrial septal aneurysm	15 (15%)	14 (11%)	0.4
Patent foramen ovale	16 (16%)	12 (9%)	0.1
Aortic arch atherosclerotic plaques	1 (1%)	3 (2%)	0.6
Carotid artery non-stenotic plaques	59 (59%)	76 (60%)	1
**Atrial cardiopathy markers**			
PWD (ms)	46 ± 13	51 ± 14	**0.01**
P wave max (ms)	125 ± 15	131 ± 20	**0.01**
P wave min (ms)	79 ± 15	81 ± 20	0.7
P wave mean (ms)	103 ± 13	107 ± 18	0.05
P wave index (ms)	15 ± 5	16 ± 5	**0.01**
PR interval (ms)	166 ± 35	171 ± 33	0.3
P wave axis (degree)	47 ± 22	50 ± 27	0.3
Left atrial area (cm^2^)	18 ± 4	20 ± 6	**0.01**
High PWD (>40 ms), *n* (%)	53 (53%)	87 (68%)	**0.02**
Abnormal P wave axis, *n* (%)	9 (9%)	27 (21%)	**0.01**
LA dilation (>20 cm^2^), *n* (%)	28 (28%)	54 (42%)	**0.03**
LA volume (ml)	48 ± 19	54 ± 24	0.1
LA volume index (ml/m^2^)	27 ± 11	31 ± 14	**0.04**

*Data are expressed as mean ± SD. CAD, coronary artery disease; PWD, P wave dispersion; LA, left atrium. Bold values are statistically significant (p < 0.05)*.

The presence of non-stenotic (<50% diameter stenosis) atherosclerotic carotid artery plaques was also similar in both groups.

Moreover, there were no differences between patients with severe and mild ESUS regarding minor-risk potential embolic sources, except for mitral valve calcifications (40% of patients in severe ESUS vs. 22% in mild ESUS; *P* = 0.004).

### Markers of Atrial Cardiopathy

Patients with severe ESUS had higher values of PWD (51 ± 14 vs. 46 ± 13, *P* = 0.01), P max (131 ± 20 vs. 125 ± 15 ms, *P* = 0.01), and P wave index (16 ± 5 vs. 15 ± 5 ms, *P* = 0.01). The number of patients with increased PWD (>40 ms) was higher in severe ESUS than in mild ESUS (87/127 vs. 53/99; *P* = 0.02). Furthermore, an abnormal P wave axis was detected more frequently in severe stroke patients (21 vs. 9%, *P*: 0.01).

Left atrial size was significantly increased in severe ESUS in comparison with mild ESUS in terms of area (20 ± 6 vs. 18 ± 4 cm^2^, *P* = 0.01) and volume (31 ± 14 vs. 27 ± 11 ml/m^2^, *P* = 0.04); in particular, the percentage of patients with increased left atrial area (>20 cm^2^) was higher in severe ESUS than in mild ESUS (54/127 vs. 28/99; *P* = 0.03) and, similarly, the number of patients with moderate-severe LA dilation (≥ 30 cm^2^) was significantly higher in severe than in mild ESUS (10/127 vs. 1/99; *P* = 0.02) (Table 1).

### Multivariate Analysis

**Figure 1:**
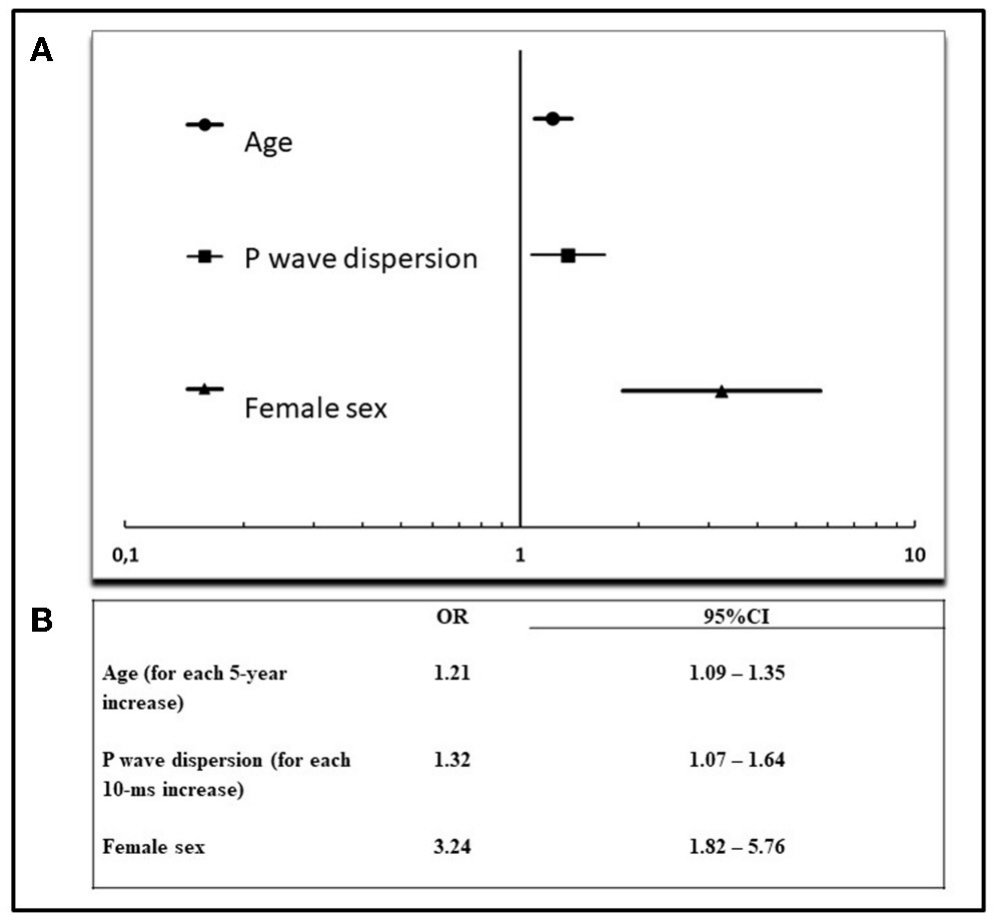
Multivariate analysis of factors associated with severe ESUS. **(A)** Forest plot of significant factors associated with severe ESUS (according to Akaike's information criterion), **(B)** results of multivariate analysis. OR, odds ratio; CI, confidence interval.

Multivariate logistic regression model was constructed to determine the factors associated with severe ESUS. The following variables were initially evaluated: age, sex, PWD, P-max, P-mean, P index, PQ interval, abnormal P axis, left atrial area, left atrial volume index, atrial dilation (left atrial area >20 cm^2^), moderate-severe atrial dilation (left atrial area ≥30 cm^2^), MV disease. Akaike's information criterion showed that the best subset of associated variables for severe ESUS were the following: age (OR = 1.21 for each 5-year increase, 95% CI: 1.09–1.35), sex (OR = 3.24 for female sex, 95% CI: 1.82–5.76), PWD (OR = 1.32 for each 10 ms increase, 95% CI: 1.07–1.64) (Figure 1).

## Discussion

The novel findings of our study are the following: there is a significant association between markers of atrial cardiopathy and severe ESUS, and this association, specifically for PWD, is independent of other clinical characteristics such as age and sex.

Indeed, in patients with severe ESUS we found the presence of both electrocardiographic and echocardiographic markers of atrial cardiopathy, including an increased P wave dispersion, P wave index, a higher rate of abnormal P wave axis, an increased left atrial size and a higher frequency of mitral valve calcifications in comparison with mild ESUS patients. These markers besides being atrial cardiopathy markers, also represent significant predictors of AF. Indeed, previous studies demonstrated that left atrial volume index is associated with new-onset AF and stroke recurrence in ESUS patients (17), many electrocardiographic markers of atrial cardiopathy can predict AF occurrence (18) and mitral valve calcification, even when asymptomatic, is associated with increased risk for AF (19) and stroke (20).

These findings suggest that atrial cardiopathy can represent the pathogenic mechanism underlying severe ESUS, while in mild ESUS other pathogenic mechanisms need to be explored.

Moreover, patients with severe ESUS are also characterized by additional and specific clinical characteristics, such as older age and higher prevalence of female sex, according to a previous study (6), that demonstrated that women have more severe ESUS compared with men.

The identification of possible cardioembolic mechanisms underlying severe ESUS could be particularly important to perform an appropriate therapy in this clinical subtype of ESUS where atrial cardiopathy could cause cardioembolism by itself or by promoting atrial fibrillation. The hypothesis that many ESUSs were determined by cardioembolic mechanisms based on silent and paroxysmal atrial fibrillation has been the rationale of RESPECT-ESUS and NAVIGATE trials (21, 22), that, however, didn't demonstrate the superiority of anticoagulants in ESUS. Nevertheless, a subanalysis of the NAVIGATE trial suggested a beneficial effect of anticoagulant therapy in ESUS associated with increased left atrial size (atrial diameter ≥46 mm) (23), confirming a possible pathogenic role of atrial cardiopathy in these patients.

Among various electrocardiographic and echocardiographic markers of atrial cardiopathy, our multivariate analysis showed that PWD represents the most significant atrial marker associated with severe ESUS.

Previous studies demonstrated that high PWD values correlate with both atrial cardiopathy and higher risk of AF, regardless of the increased atrial size (24–27). In this view, high PWD (even in presence of a normal left atrium size) represents an index of atrial electrical heterogeneity, reflecting the presence of atrial microarchitecture change and site-dependent conduction delay, also due to inflammatory mechanisms, that can favor AF occurrence (9, 28, 29).

Our results are particularly important because they suggest that severe ESUS patients are characterized by higher risk of atrial cardiopathy. However, even if atrial cardiopathy is a condition that encompasses alterations in macrostructure and microstructure that are associated with ischemic stroke also independent of atrial fibrillation occurrence (30), current evidence doesn't suggest the efficacy of anticoagulant therapy in ESUS only associated to atrial cardiopathy without identification of AF episodes (31). For this reason, an intensive ECG monitoring is particularly important to detect subclinical AF and to choose the most appropriate therapy in this subgroup of ESUS (32).

Furthermore, the ongoing ARCADIA trial (33), comparing apixaban and aspirin in ESUS associated with atrial cardiopathy, will provide further data about the possible beneficial effects of anticoagulants in this subgroup of ESUS, also regardless of AF occurrence.

Our study has some limitations. The recruitment was mono-centric and we have no data about the ECG monitoring in the long-term, even if previous studies confirmed PWD as a predictor of AF after ischemic stroke in short- and long-term (18, 24, 34).

Furthermore, although ESUS is usually characterized by mild symptoms, most of our patients were affected by severe ESUS, because our Stroke Unit is a Comprehensive Stroke Center, that usually admits and treats the most complex and severe strokes from the entire South-East Tuscany.

Further studies are necessary to better characterize clinical characteristics of ESUS patients, to identify different pathogenic mechanisms underlying various subtypes of ESUS.

## Conclusions

Our findings shed light on specific clinical characteristics of severe ESUS including the presence of atrial cardiopathy that could play a pathogenic role in this subgroup of patients. Searching for atrial fibrillation in these patients is especially important in order to perform the most appropriate therapy.

## Data Availability Statement

The raw data supporting the conclusions of this article will be made available by the authors, without undue reservation.

## Ethics Statement

The studies involving human participants were reviewed and approved by Ethics Committee of the University Hospital of Siena, Italy. The patients/participants provided their written informed consent to participate in this study.

## Author Contributions

MA conception and design of the work. MA, PL, CD, FG, RT, and GM substantial contributions to the acquisition of data for the work. MA, PL, and AC substantial contributions to the analysis of data for the work and drafting the work. MA, PL, GM, and AC substantial contributions to the interpretation of data for the work and revising the draft of the work critically for important intellectual content. MA, PL, FG, RT, CD, GM, and AC final approval of the version to be published and agreement to be accountable for all aspects of the work in ensuring that questions related to the accuracy or integrity of any part of the work are appropriately investigated and resolved. All authors contributed to the article and approved the submitted version.

## Conflict of Interest

The authors declare that the research was conducted in the absence of any commercial or financial relationships that could be construed as a potential conflict of interest.

## Publisher's Note

All claims expressed in this article are solely those of the authors and do not necessarily represent those of their affiliated organizations, or those of the publisher, the editors and the reviewers. Any product that may be evaluated in this article, or claim that may be made by its manufacturer, is not guaranteed or endorsed by the publisher.
